# Process development of ATROSAB, an anti TNFR1 Monoclonal Antibody: in three steps from research to GMP

**DOI:** 10.1186/1753-6561-5-S8-P42

**Published:** 2011-11-22

**Authors:** Karlheinz Landauer, Cuneyt Unutmaz, Simon Egli, Verena Berger, Simone Lais, Timo Liebig, Daniel Steiner, Jo Maier, Ina Rostalski, Francois Forcellino, Andreas Herrmann

**Affiliations:** 1 Celonic AG, Basel, Switzerland; 2 Celonic GmbH, Jülich, Deutschland

## 

The humanized monoclonal antibody ATROSAB is targeting specifically the TNF receptor 1(TNFR1). TNF is a central mediator of inflammation and key target for intervention in inflammatory diseases such as rheumatoid arthritis, psoriasis and Crohn’s Diseases. Notably, blockade of the second TNF receptor, TNFR2, has been associated with increased sensitivity to viral infections or increased susceptibility to demyelinating disorders and lymphomas. In this context, a selective inhibition of TNF-induced TNFR1 but not TNFR2 signaling activity holds great promises to overcome undesired effects observed with less specific TNF antagonists currently used in clinic.

The recently humanized antibody was used to establish a rCHO-K1 cell line under serum-free, chemically defined conditions (1). The process development was based on two sets of shake flask experiments, three 10L bioreactor runs and finally 2 GMP production runs in 300 L scale. The scale up strategy was based on quality by design considerations specified in the design space for the scale-up parameters. The main parameter for the scale-up development was mixing time as a function of the bioreactor geometry, tip-speed, Reynolds number, and the power input of the systems. Those parameters are generally considered as important parameters during up-stream process development.

Protein characteristics were specified based on several considerations. A major part was the ICH-Q8 guideline as well as antibody and other drug guidelines. The other part is based on experience with antibody development and the specific protein in special. The specifications are described in Table 1. The analytical methods used to characterize the antibody were SDS-PAGE, western blot, HPLC-SEC, protein A HPLC, HPAEC-PAD, Isoelectric focusing and a cell based potency assay. As the antibody is declined to bind to the TNF-R1 on target cells, it was engineered not to elicit ADCC or CDC. Therefore those parameters were not checked during process development.

**Table 1:** specified scale-up parameters and defined protein characteristics

	scale-up parameter		protein characteristics	
**Parameter**	**Design Space**	**300L GMP**	**specifications defined at the project start**	**300L GMP**

Mixing Time [s]	10 - 70	max. 60	Isoforms (IEF)	pH 7.6 - 8.8
Tip-Speed [m/s]	0.6 - 1.2	max. 1.0	Glycosylation (HPAEC-PAD)	Mol% of different forms ± 20% from clone screening
Reynolds Number	1 - 8 Mio	max. 6 Mio	Potency	40% - 160% from clone screening
Power Input [W/m3]	25 - 1100	max. 650	Molecular weight (SDS-PAGE)	Main Band at app. 150 kDa
Gassing	Head Space / Ring Sparger	Head Space & Ring Sparger	Immunogenic glycostructures	none
Agitation	orbital sh., Rushton I.	Rushton I.	Purity (HP-SEC)	> 90%

The basal medium was fixed to a commercial available chemically defined medium during clone screening. In a batch process, performed in a fully equipped 1L bioreactor, a titer of approximately 180 mg/L was obtained. At this stage the antibody was characterized and specifications were partly narrowed and some were newly set. In the first step of process development different feeding solutions and concentrations thereof were tested in shake flask experiments. Metabolite data were monitored on a daily basis comprising glucose, lactate, ammonia, and amino acid levels. Additionally cell density and protein concentration were measured at the same time interval. For in-depth protein characterization, supernatant was purified after 6 days of fermentation and at harvest, in order to check the quality attributes already early at the development stage. This step led to an increase of about 2.5 fold in product concentration, while the protein characteristics stayed within specifications at both sampling time points. The second step was the optimization of the feeding strategy as well as the fortification of the solutions with certain chemically defined supplements. This led to another increase of about 2.5 fold regarding final product concentration. Product quality changed slightly, but stayed within specifications. Especially the glycosylation structure changed towards less glycosylated versions. However, no impact on the bioassay was observable. The process was finally scaled up to the 300L stirred tank bioreactor in the GMP facility. An engineering run was performed to test the process and product parameters. Some influence was measured, thus the process was slightly changed prior the first GMP run. The obtained product quality was well within specifications, the achieved titer was more than 1.2 g/L, which was a more than 7 fold increase in comparison to the initial batch process. Although the cell density profile of the process changed between run 1 and 2, the volumetric product concentration was only slightly below 1.2 g/L and the product characteristics were identical to the first run. This indicates the robustness of the developed process and the validity to define and specify design spaces for process development especially during scale-up development. The analysis of protein characteristics employing SDS-PAGE, IEF, HP-SEC, potency assay and glycosylation pattern with HPAEC-PAD ensures the quality of the protein.

**Figure 1:**
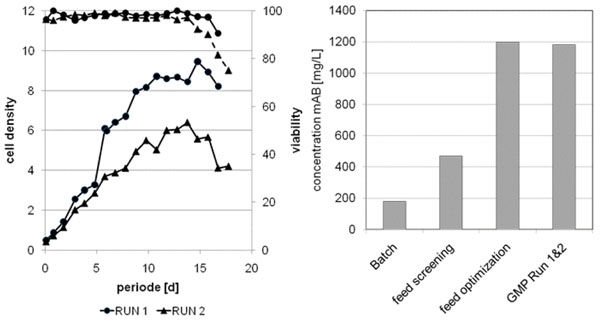
The left diagram shows the cell density of two GMP runs in the 300L bioreactor. The right diagram focuses on the achieved product concentrations during the different process development steps. The batch experiment was performed in 1L bioreactors, the feed screening was done in 250 mL shake flasks. The feed optimization was performed in 10L bioreactors and the GMP runs were done in a 300L stirred tank bioreactor in a GMP facility.
